# Uncertainty analysis of turbine nozzle guide vane cooling performance

**DOI:** 10.1371/journal.pone.0324310

**Published:** 2025-05-28

**Authors:** Decang Lou, Mengjun Wang, Along Chen, Jun Zeng, Shuai Zhang, Xiaoyang Huang

**Affiliations:** 1 Department of Power and Energy, NorthWestern Polytechnical University, Xian, Shanxi, China; 2 AECC Sichuan Gas Turbine Research Establishment, Chengdu, Sichuan, China; King Fahd University of Petroleum & Minerals, SAUDI ARABIA

## Abstract

Conjugate heat transfer analysis plays an important role in the design of heavily cooled gas turbine vane or blade. In this study, the aerodynamic and heat transfer performance of the E3 engine nozzle guide vane is investigated through conjugate heat transfer analysis using ANSYS CFX software. Computational fluid dynamic analysis indicates that the shear stress transport turbulence model gives high accuracy in simulating the vane main-stream aerodynamic performance. However, when the simulated surface temperature of the vane was compared with the cooling test data, in which the vane was 3D-printed, significant deviation of the surface temperature distribution was observed. To understand the sources of these deviations, analyses are conducted considering main-stream and coolant flow parameters, as well as manufacturing discrepancies. The results reveal that the large deviation in the manufactured vane (up to 0.5 mm at the leading edge) alters the direction of the coolant flowing out from the leading-edge film-cooling holes, affects the film coverage along the surface, and in consequence, causes the temperature near the stagnation point increasing by approximately 40 K. Furthermore, variations in coolant inlet pressure, decreasing by 10 kPa, and temperature, increasing by 10 K, result in the vane surface temperature increased by 20 ~ 30 K. When the turbulence intensity of the main-stream increased from 5% to 20%, the vane surface temperature increased by approximately 20 K. Hence when conducting conjugate heat transfer analysis to validate the cooling performance of turbine vane or blade, emphasis should be focused on not only the uncertainties of the aerodynamic parameters but the manufacturing deviations.

## 1. Introduction

The efficiency of the gas turbine cycle increases as the turbine inlet temperature (TIT) increased. In the long and complex evolution of gas turbines, the TIT has risen from about 1050K in 1944 to about 1750K in the 1990s as the turbine of Energy Efficient Engine (E3) being designed. In the future the TIT may exceed 2300 K, which is far beyond the allowable working temperatures of turbine components, such as guide vanes, blades, and disks [1,2]. Therefore an effective cooling design is essential for reducing the wall temperature of the turbine vane [3]. The blade cooling technology have developed from the initial simple passage in 1960s to the complex combination of convection, impingement and film cooling structures in 1990s [4]. In this development process, numerical simulation plays an important role. However, for a heavily cooled guide vane or blade of high pressure turbine (HPT), conjugate heat transfer simulation is one of the most challenging topics in the computational fluid dynamics (CFD) field [5]. It serves as an important tool for designing turbine cooling systems, and the accuracy of temperature field prediction is crucial for predicting turbine vane lifespan and cooling structural design. Up to now, numerous comparative studies have been based on the experimental data from MARK II and C3X blade cascades. The computational work by York and Leylek [6], Takahashi et al. [7], Mazur et al. [8], Ho et al. [9], Zeng and Qing [10,11], Wang et al. [12], and Zhou et al. [13] demonstrated that the k-omega shear stress transport (SST) model with transitional turbulence provides high accuracy in simulating the pressure distribution, turning position, temperature, and external heat transfer coefficient on the surface of the turbine vane grille at subsonic and transonic speeds. Moon and Kim [14] established the relationship between the heat transfer and flow resistance coefficients against the Reynolds number for a novel fan-shaped pin-fin within the internal cooling channel of a turbine blade, using a low Reynolds number SST model, and applied the findings to the cooling design of turbine blades. Al-Obaidi et al. [15] also discovered that the numerical results with SST (k-ω) turbulence model of an axial flow pump exhibit a commendable alignment with the experimental data. Extensive studies [16–21] were conducted to studied the effects of varying flow conditions, inlet pressure pulsation, swirling direction, different guide vane configuration and blade angle on pump’s main flow characteristics, vortex and dynamics. They found that the mainstream characteristics of the pump heavily relying on the inlet flow conditions and configurations. Zeng et al. [22] performed a conjugate heat transfer (CHT) analysis through numerical simulation for a hollow, air-cooled, low-pressure turbine dynamic lobe, comparing the results with blade cooling efficiency test data; they found a deviation of only 0.3% between the calculations and test data. Kukutla et al. [23,24] studied the interaction between the hot mainstream and the coolant effusing out from the showerhead film holes of NGV with both the PIV experiment and the simulation. The mass flow rate of the coolant has great effect on the pressure drop, temperature rise and hot gas ingestion of the coolant. In conclusion, CHT analysis plays an important role in turbine cooling design and the k-omega SST turbulence model predicts heat transfer precisely for turbine vane or blades.

However, most of the conjugate heat transfer in existing literature focus on simple cooling structures. For advanced, high-efficiency cooling blades featuring complex cooling configurations, relevant test data are scarce. Before the CHT methods were extensively validated for complex cooling vanes, cooling effectiveness tests are still necessary to prove the design. Although some literatures [25,26] gave the test results, most of them only presented the data without detailed geometric information. The E3 engine nozzle guide vane has detailed modelling and experimental information. Hence in this study, it was selected as the investigation object. The CHT analysis was firstly conducted to study the heat transfer performance of the E3 engine nozzle guide vane with complex cooling structures. After the CFD model was validated with literature reported aerodynamical data, a cooling effectiveness experiment was then carried out to obtain the NGV surface temperature distribution. To discover the cause of the discrepancies between the CHT results and the cooling effectiveness test, a comprehensive uncertainty analysis was conducted. Various influential factors including the inlet turbulence intensity of the main stream, deviations in the temperature and pressure of the coolant, and profile discrepancies between the manufactured and nominal NGV were quantitatively studied. Investigation results were helpful to quantify the uncertainty of the turbine nozzle guide vane cooling performance.

## 2. Numerical/experimental methodology

In this section, the geometrical model of the E3 engine nozzle guide vane was built up firstly. Then the computational fluid dynamic models including both mesh and turbulence model were validated with the reported data. In addition, the experimental methodology for cooling performance of NGV was described.

### 2.1. Model of E3 engine nozzle guide vane

**Fig 1:**
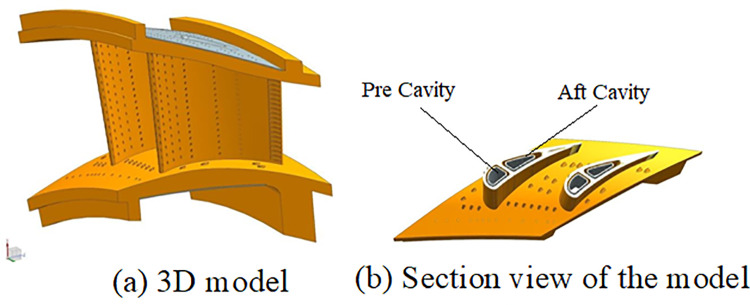
3D model and section view of E3 NGV.

**Fig 2:**
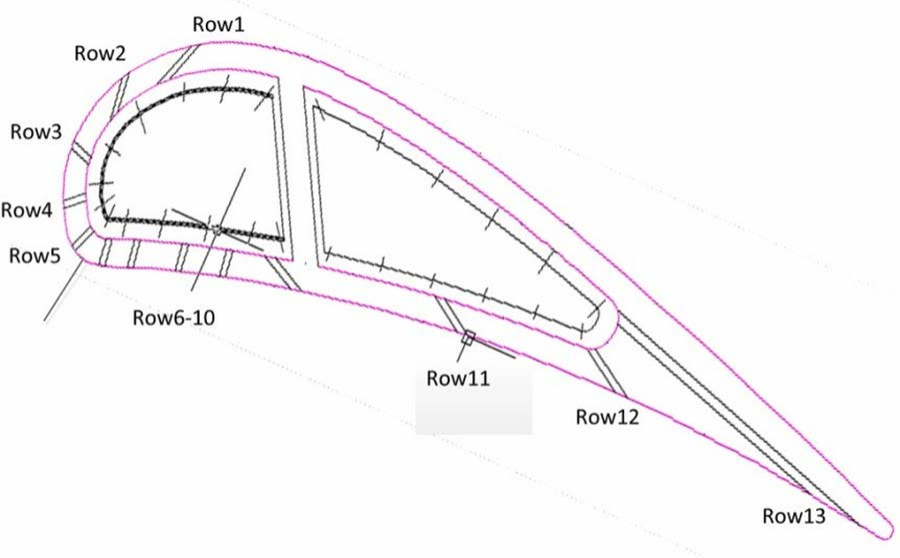
Distribution of film cooling holes along the section of NGV.

The detailed design and analysis results of the high-pressure turbine nozzle guide vane (NGV) for the E3 engine were documented by Halila et al. [27]. Fig 1 illustrates the 3-D model (Fig 1a), section view (Fig 1b) of the NGV, and Fig 2 gives the distribution of film cooling holes along the its section. The NGV is divided into two cavities by a rib, which slants aft from tip to hub to maximize the flow entrance area. Cooling air for both the pre and aft cavities comes from the inner and outer combustor liners, respectively. A combination of film and impingement cooling has been employed at both the leading edge and the mid-portion of the vane, with convective cooling being applied to the trailing edge. There are totally 13 rows of impact holes on the pre-cavity insert. The cooling air flows through these impact holes and then was injected into the mainstream through 10 rows of film cooling holes. For the aft cavity, there are 12 rows of impact holes on the aft insert, 2 rows of film cooling holes on the pressure side and one slot on the trailing edge. The cooling air at the pressure side is partly injected into the mainstream by film cooling holes to form impact-film cooling effect, and the remaining flow through the slots at the trailing edge.

### 2.2. Validation of CHT analysis for the nozzle guide vane

**Fig 3:**
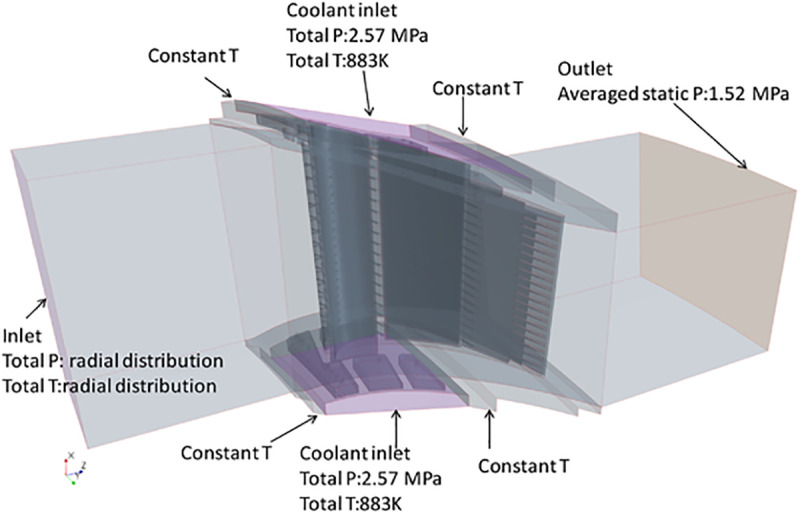
Boundary conditions for high-pressure turbine guide vane.

In this project, the precision of the CHT analysis is very important. Hence extensive studies including the mesh, turbulence model, boundary and the convergence step have been conducted to ensure the repeatability and precision of the CHT models. The conjugate heat transfer analysis is carried out for the NGV using the ANSYS CFX software package for simulation. The boundary conditions are listed in Fig 3. The given total temperature and total pressure at the turbine inlet are distributed along the radial direction according to the combustor outlet profile [28], with an average total pressure of 2.53 MPa and an average total temperature of 1742 K. The inlet pressures of the coolant for the leading-edge and aft cavities are 2.57 MPa, respectively. Moreover, the total temperature of the coolant for both cavities is set at 883 K. The back pressure is set at 1.52MPa.

The k-w shear stress transport (SST) turbulence model was applied while the γ-Reθ transition model was turned on. This model was developed by F.R.Menter for better predicting the transition from laminar to turbulent. The detailed mathematic expression for the model can be found in reference [29]. The SST turbulence model works well for external aerodynamics, such as airfoils turbine vanes, and for internal flows, such as cooling passages. It can accurately capture the boundary layer separation and reattachment which (important for airfoils, turbine blades) and provides better predictions than standard k − ω or k−∊. However, it requires fine near-wall meshing (low y+ values ~1) to achieve high accuracy.

**Fig 4:**
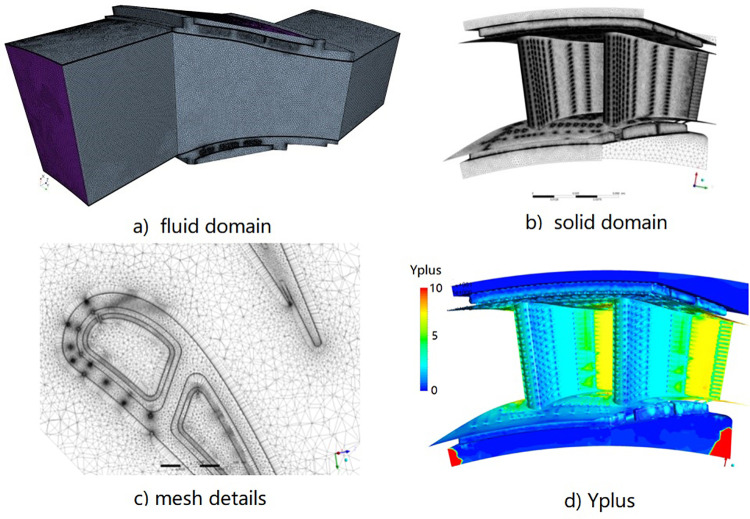
Mesh grid for both fluid and solid domains. a) Fluid domain, b) solid domain, c) Mesh details, d) Yplus.

High quality mesh is critical in the CHT analysis. Mesh convergence studies have been conducted firstly. Three mesh schemes are implemented as illustrated in Table 1. As an example, the mesh for both fluid and solid domain (Mesh 3) is shown in Fig 4. There are totally 62.85 million elements for the fluid domain (Fig 4a) and 37.66 million elements for the solid domain (Fig 4b). Boundary layer was set on both the fluid and solid domain (Fig 4c). The first layer thickness was set 2.0e-7m on each side and the growth rate was 1.2 and 1.3 for fluid and solid domain respectively. To get a better result with the shear stress transport (SST) turbulence model, the y-plus values along the surface of both the vane and the inner cavity are around 1. The detail mesh around the wall is shown in Fig 4c, where the maximum values of y-plus (Fig 4d) are less than 10 at some high speed areas.

**Table 1:** Scheme for mesh convergence study.

	Mesh1	Mesh2	Mesh3
Fluid domain, million	9.94	50.17	62.85
Solid domain, million	2.79	20.35	37.66

**Fig 5:**
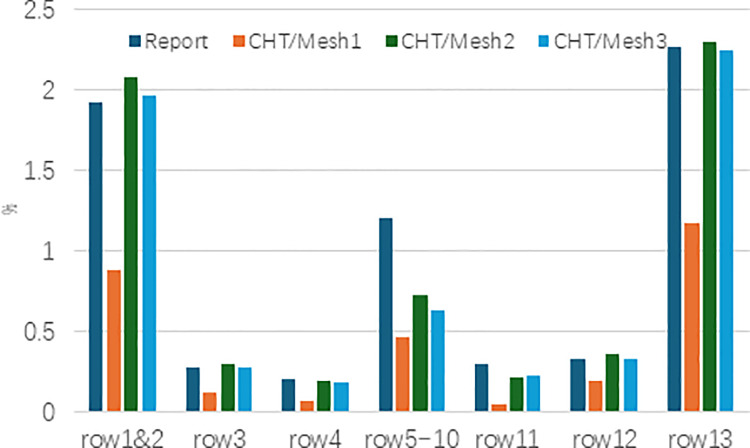
Coolant flow rate for different rows of film-cooling holes.

Fig 5 compares the CHT simulated coolant flow rate for each row of film-cooling holes under different mesh schemes with the reported data [27]. In the figure, the vertical coordinate represents the percentage of mass flow rate relative to the compressor inlet mass flow rate (W25) of the engine, and the axial coordinate refers to the row number of the film-cooling holes. It shows that the coolant mass flow rate converged to the reported data with the increasing of the mesh count. Finally Mesh3 is selected for further analysis due to its better agreement with the documented data.

**Fig 6:**
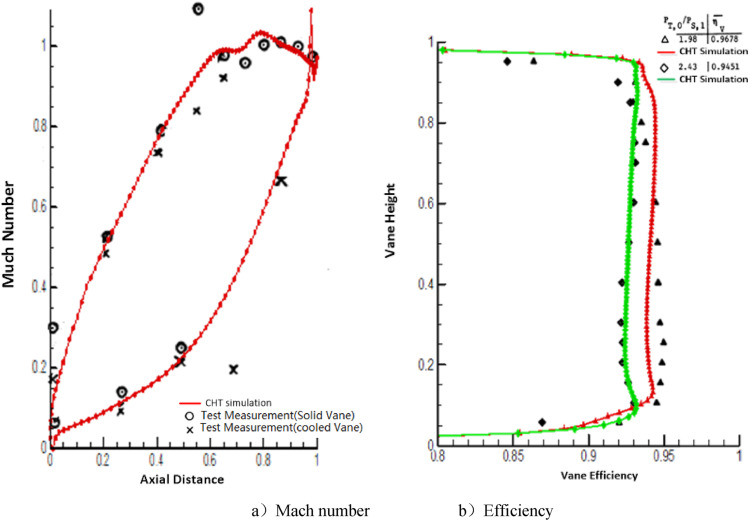
Comparison between CHT simulation and test data [28].

Fig 6 shows that the distributions of the simulated Mach number (Fig 5a) and efficiency (Fig 5b) for the high-pressure turbine NGV align with the experimental data from the literature [28]. This suggests that the chosen software and turbulence model provide a high level of accuracy (less than 1.5% for efficiency) for simulating the main-stream parameters of the turbine vane cascades.

Table 2 compares the simulation results with those values listed in NASA report [28]. The maximum deviation for turbine inlet mass flow, G_in_, is only 0.17%.

**Table 2:** Comparison of simulated parameters with reference data [28].

	CHT Simulation	NASA report [28,30]	diff, %
G_in_,kg/s	49.81	49.72	0.17
T^*^_in_, K	1742.74	1742	0.04
P^*^_in_, MPa	2.53	2.53	0.00
G_out_,kg/s	55.29	55.26	0.06
T^*^_out_, K	1660.56	1660	0.03

However, concerning the wall temperature distribution of the NGV, the existing literature only provides certain calculation parameters and lacks experimental data. Therefore, a detailed comparison regarding the accuracy of wall temperature calculations is not available. In this study, a cooling effectiveness experiment is conducted to obtain the surface temperature distribution, thereby providing further validation for the CHT analysis.

### 2.3. Cooling effectiveness test setup and methodology

1) **Test setup**

**Fig 7:**
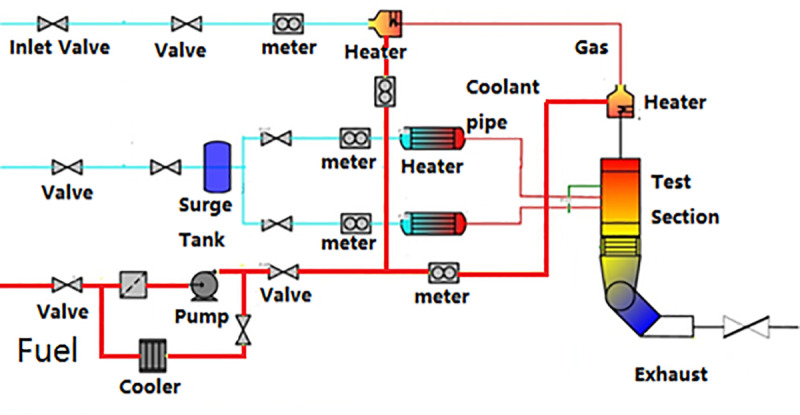
Schematic of the test equipment.

To achieve the temperature distribution of the NGV, the cooling effectiveness test was designed. The purpose of the test was to validate the cooling effectiveness and the aerodynamic performance. The schematic of the NGV cooling effectiveness test rig is shown in Fig 7. The test equipment comprises a gas inlet system, a gas heating system, a test section, an exhaust system, a fuel system, a cooling air heating system, and a test status monitoring and data acquisition system. The main gas pipeline is equipped with electric valves to control and regulate the main gas intake flow, which is measured by a flow nozzle meter. A two-stage heater is utilized to heat the main gas to meet the test requirements. An electric butterfly valve is installed in the exhaust section to ensure the test meets the specified pressure drop ratio requirement. A high-precision flow meter is fitted to each cooling air pipe to measure the coolant air flow. Each cooling air pipe is equipped with a high-power stainless steel electric heater. Measuring devices for total pressure, static pressure, and total temperature are positioned at the inlet and outlet of the test section. At the same time, the inlet temperature and total pressure for the coolant were measured.

2) **Test methodology**

**Fig 8:**
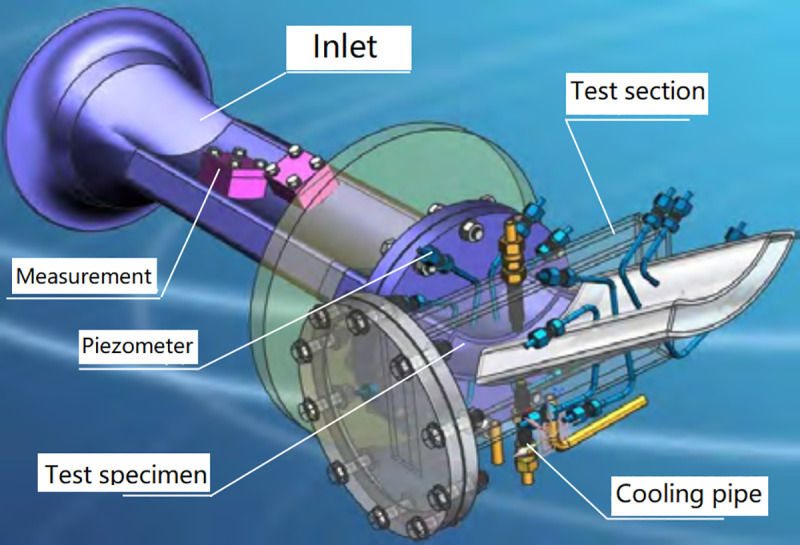
Scheme of the test section.

**Fig 9:**
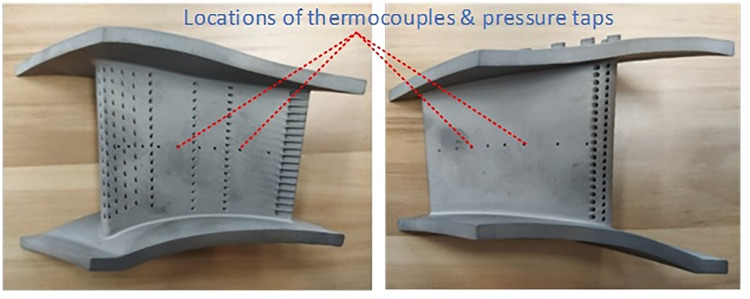
3-D printed NGV specimen.

Fig 8 shows scheme of the test section which is mounted in a compartment and secured with bolts in the circumferential direction [30]. In this study, the NGV test specimen was 3-D printed with Ni-based alloy (IN718). As was shown in Fig 9, the NGV wall surface temperature is measured with thermocouples which were located along its middle section. The thermocouples with diameter of 0.5mm, were fixed inside slots, which were 0.55 mm in depth and width, under the vane surface. The type K thermocouple, VRKK-102/II type, was used for temperature measurement and its error was 1K. The error for the pressure measurement was 1%.

During the cooling effectiveness test, several non-dimensional parameters should be kept constant with the designed value to maintain similarity of the flow and heat transfer of the NGV. Here three main parameters are given, including the mass flow ratio, pressure ratio and the temperature ratio. Equation (1–3) show the definition of these parameters which can be found in reference [30].

The mass flow ratio is expressed as equation (1):


$$\begin{array}{c} K_{W}=\frac{W_{C}}{W_{g}} \end{array}$$ (1)

In which $W_{C}$ is the coolant mass flow rate of the test specimen. $W_{g}$ is the NGV inlet mass flow rate of gas side.

The pressure ratio is expressed as equation (2):


$$\begin{array}{c} \pi_{T}=\frac{P_{t\_in}}{\overset{―}{P_{s\_out}}} \end{array}$$ (2)

In which, Pt_in, Ps_out were the inlet total pressure and the outlet static pressure of the NGV gas side respectively.

The temperature ratio is expressed as equation (3):


$$\begin{array}{c} K_{T}=\frac{T_{t\_in}}{T_{C\_in}} \end{array}$$ (3)

Where Tt_in and $T_{C\_in}$ were inlet total temperature of NGV for gas side and coolant side respectively.

3) **Test case**

The test parameters for the NGV were listed in Table 3. CHT simulation was conducted to study the flow and heat transfer performance under the test conditions. The type of the boundary conditions is same as that described in section 2.2. In the vane cooling test, the mainstream inlet pressure and temperature are set at 900kPa and 1000K respectively. And the pressure ratio, temperature ratio and mass flow ratio are same as that design condition.

**Table 3:** Main test parameters.

Parameter	Unit	value
Pressure ratio		1.7
Temperature ratio		1.9
Mass flow ratio	Percentage	8.0

The numerical and experimental methodology built up in this section will be employed to study the NGV cooling performance. The object of the CHT simulation is to obtain the accuracy of the simulation and found the influence of different parameters.

## 3. Results and discussions

In this section, the cooling effectiveness experimental results of NGV were firstly illustrated, and the CHT simulation results were displayed as comparison at the same time. Then the differences between simulation and experiment were discussed. Influential factors including the main stream inlet turbulence intensity, deviations in the temperature and pressure of the coolant, and profile discrepancies between the manufactured and nominal NGV were quantitatively studied.

### 3.1. Results of the cooling effectiveness test

**Fig 10:**
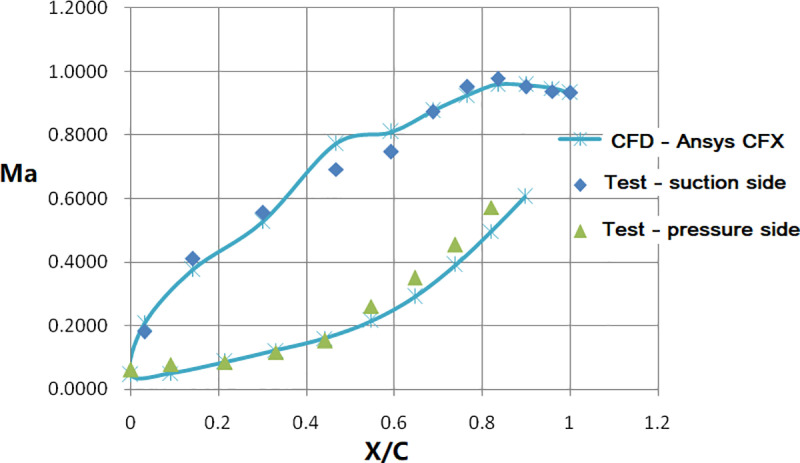
Comparison of the isentropic Ma distribution along the middle section of NGV.

**Fig 11:**
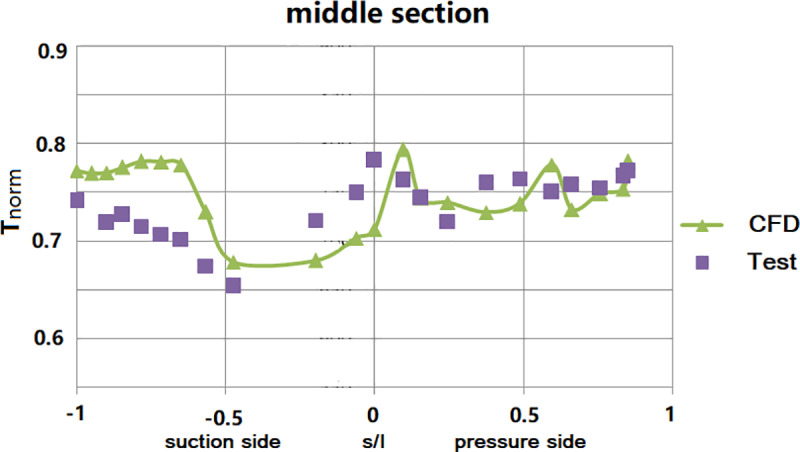
Comparison of surface temperature distribution along the middle section of NGV.

**Fig 12:**
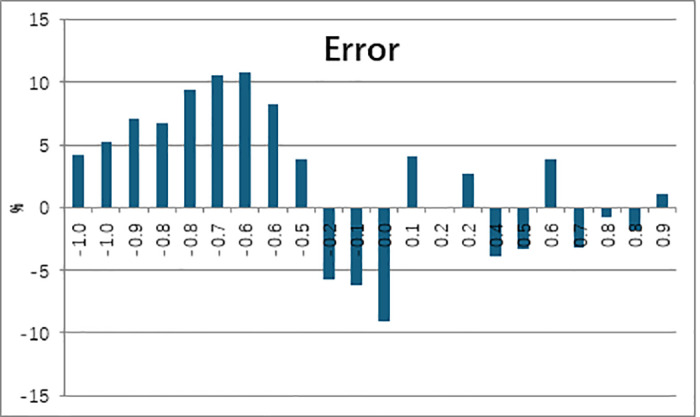
Relative error bar of surface temperature.

Fig 10 illustrates the comparison between the simulated isentropic Mach number distribution on the vane surface and the test data. The simulated Mach number distribution on both pressure and suction sides aligns well with the test data. Fig 11 compares the simulated surface temperature distribution of the NGV middle section (normalized with the inlet total temperature) with the measured data. It shows that there exists two obvious difference. Firstly, at the leading edge, there exists a positional deviation in the local temperature distribution between the simulation and test. the simulated maximum temperature, in the leading-edge area, locates at the pressure side of the NGV, while it was at the stagnation point during the test. Secondly, the simulated temperature at the trailing edge on the suction side of the NGV is higher than the measured value. The error bar of surface temperature with CHT simulation comparing to experiment was shown in Fig 12. The maximum relative error for the CHT simulation can reach 11% at the middle section of the suction side. At the leading edge of NGV, the error was near -10%. For the pressure side, the relative error was under 5%.

**Fig 13:**
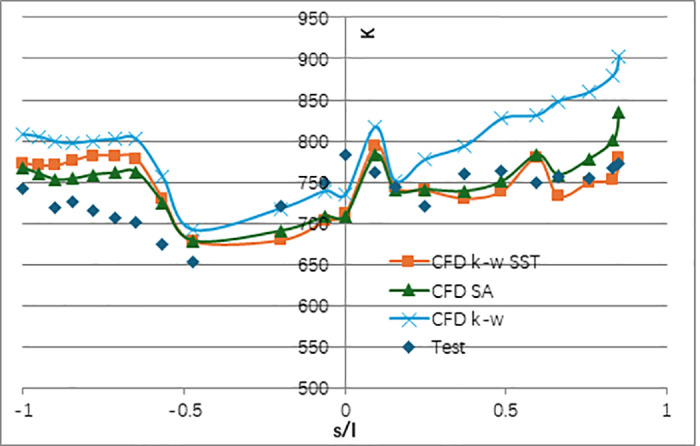
The temperature difference between the SA model and the k-w SST model.

Before we conducted further uncertainty analysis, the influence of turbulence model including the K-w SST, SA, and k-w, was firstly studied. The temperature distributions for the middle section of the vane are compared in Fig 13. Results show that the k-w turbulence model gives quite different temperature distribution with the experimental data. The SA model gives similar temperature distribution with the k-w SST model except overestimating at the trailing edge on pressure side. In consequence, the k-w SST turbulence was selected in the following comparison.

During the test, the probe of the thermocouples was embedded 0.55mm underneath the surface. Simulation results show that temperature difference across the wall is around 10K. The wall thickness for most part of the vane is around 2mm. it can be estimated that the installation depth of the prob may introduce about 1-5K difference from the surface. In addition, due to the dense arrangement of film cooling holes at the leading edge of NGV, there was no more place for embedding the thermocouples. If the full surface measurement techniques were employed, more detailed information of temperature can be achieved.

### 3.2. Uncertainty analysis of the NGV surface temperature

As described above, significant deviations exist between the measured temperature and the numerical simulation results in the NGV cooling effectiveness test. Uncertainties that caused the deviations may arise from a variety of factors, including the uncertainty of the mainstream and coolant boundary conditions, manufacturing deviations, measurement errors and so on.

With respect to mainstream boundary conditions, there are six probes located in front of the NGV to measure the inlet pressure and total temperature. Test data shows that the values of the six probes are almost same. However, the existence of the probes may introduce the turbulence intensity. Hence the primary influential factor under consideration was the main stream turbulence intensity.

The uncertainty of the coolant side boundary conditions was mainly caused by the location of the probes on the coolant pipe for both the pressure and the temperature. There exists a distance about 200 mm between the location of probes and the vane coolant inlet cavity. Simulation results show that the distance may cause the pressure drop of about 10kPa from the measurement point to the NGV inlet cavity. It may also bring temperature rise of about 20K due to the pipe being surrounded by the leaked hot gas from the mainstream.

In terms of manufacturing deviations, 3D-printing can bring deviation of surface roughness, film hole diameter and the surface coordinates from the design. Due to the NGV’s surface being polished after the thermocouples were installed, the influence of surface roughness can be minimized. The diameter of the film holes was also inspected with the plug gauge. The maximum deviation was within 0.05 mm, which was acceptable. Finally, the vane surface coordinates were measured with a coordinate measuring machine (CMM). In this paper the vane’s profile was recreated based on the measured data to assess the impact of manufacturing deviations.

In addition, there are also other factors like turbulence model, measurement errors and so on. The CFD turbulence model have been validated in the previous section, and the measurement probes have been calibrated before the test. Hence, they are not considered for further analysis here.

Based on the above analysis, the main influential parameters are the mainstream turbulence intensity, the deviation of coolant inlet pressure, temperature and surface coordinates. To quantify and verify the effect of each factor, only one factor changes during the simulation. In the following section, there are totally six conditions being set to analyze the influence of these parameters, as was shown in Table 3, where UC0 serves as the baseline for analysis. Numerical simulations were performed with conditions described in Table 4.

**Table 4:** Conditions for uncertainty analyses.

Definition	Serial number	Characteristic parameters
Baseline	UC0	Inlet turbulence intensity: 5%
Coolant parameters	UC1	20K increase in total temperature for coolant inlet
	UC2	10kPa reduction in total pressure for coolant inlet
Main-stream parameters	UC3	Inlet turbulence intensity = 1%
	UC4	Inlet turbulence intensity = 10%
	UC5	Inlet turbulence intensity = 20%
Manufacturing profile	UC6	Reconstructed model based on CMM data

#### 3.2.1. Effect of deviations in coolant parameters.

During the test, the coolant inlet temperature and pressure probes were installed in the coolant air supply pipeline upstream of the NGV inlet cavity. Therefore, considering the loss along the pipeline, the coolant inlet pressure exists a deviation of about 10 kPa. Similarly, when the coolant enters the NGV, due to heat transfer from the surrounding high-temperature airflow, the coolant temperature could increase by up to 20 K.

1) **Influence of coolant inlet temperature**

**Fig 14:**
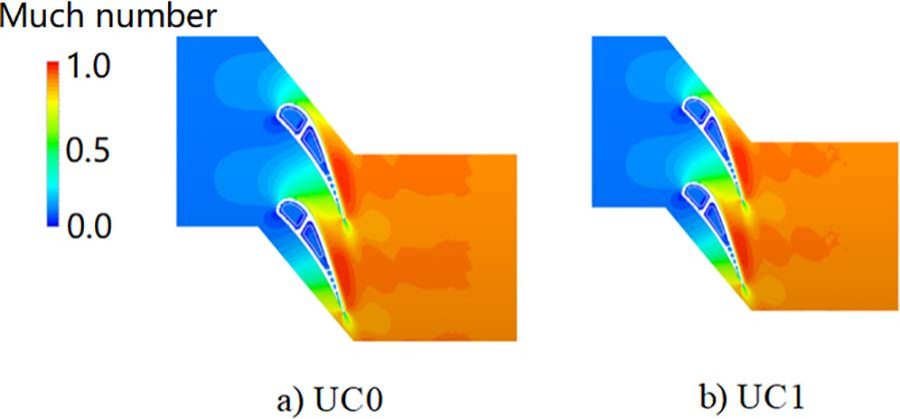
Effect of coolant temperature on the mainstream (contour of Mach number).

**Fig 15:**
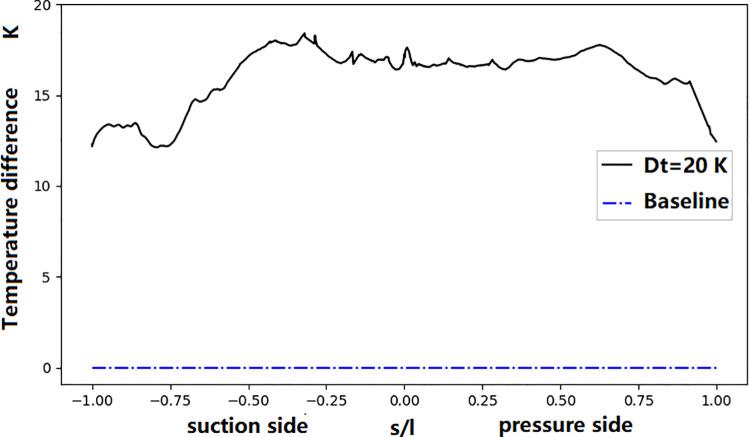
Influence of cooling temperature deviation on NGV surface temperature.

Fig 14 shows the mainstream Mach number contour of UC0 and UC1. It shows that the increase in coolant temperature has no dramatic impact on the Much number distribution. Fig 15 shows the middle section surface temperature difference between UC1 and UC0. Results show that a 20 K rise in the total coolant temperature(about 3% relative to the measured value) leads to an increase in the vane surface temperature of approximately 13–18 K. It is accompanied by a corresponding reduction in coolant mass flow; the coolant flow rates in the front and aft chambers decrease by 1.6% and 1.4%, respectively.

2) **Effect of coolant inlet pressure**

**Fig 16:**
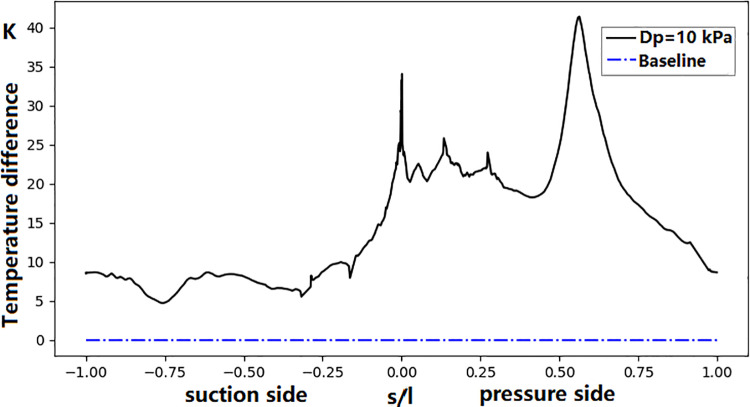
Influence of cooling inlet pressure deviation on NGV surface temperature.

When comes to the effect of the coolant inlet pressure, the 10 kPa reduction on the coolant inlet total pressure, about 1% relative to the inlet pressure, cause the coolant mass flow rate for the front and aft cavities decreasing by 10.5% and 6.2%, respectively. It has a dramatic impact on the surface temperature distribution. Due to the decrease in total coolant pressure, the minimum film-cooling hole counter flow margin (the ratio of the film-cooling hole’s inlet total pressure to the outlet static pressure) for the hole located in the front cavity decreases from 1.0289 to 1.0178. For the aft cavity film-cooling hole, the minimum counter flow margin decreases from 1.011 to 1.0, restricting coolant outflow from the film-cooling hole on the pressure side of the vane. This results in a local high-temperature region, with a local temperature increase of up to ~40 K. As was shown in Fig 16, the surface temperature increases by 20–40 K on the pressure side and by 5–10 K on the suction side when the coolant pressure decreased by 1%.

#### 3.2.2. Impact of inlet turbulence intensity.

**Fig 17:**
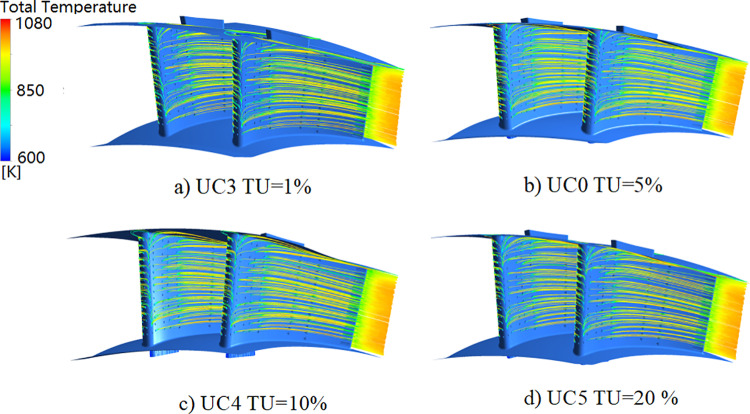
Effect of inlet turbulence intensity on the main flow (streamline diagram).

In the cooling effectiveness test, no measurement data was available for the NGV inlet turbulence intensity. To evaluate its effect, the assumed value ranges between 1% and 20% according to literature. Fig 17 presents the results of flow and temperature fields for different levels of inlet turbulence intensity (TU = 1% (Fig 17(a)), 5% (Fig 17(b)), 10% (Fig 17(c)), 20% (Fig 17(d))). Changes in inlet turbulence intensity do not significantly affect the flow field. However, as the turbulence intensity increases, the coolant flow in the front and back cavities of the vane shows a minor decrease of up to 0.3% for TU = 20%.

**Fig 18:**
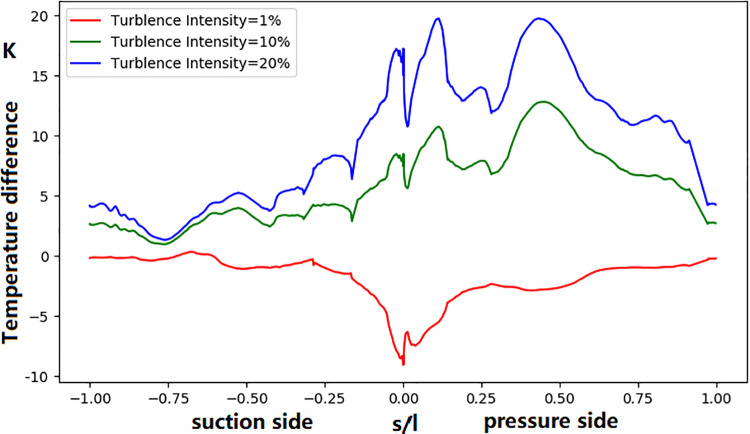
Effect of inlet turbulence intensity on NGV surface temperature (compared to baseline).

Fig 18 compares the difference of the vane surface temperature distribution with the baseline. It shows that an increase in the main-stream inlet turbulence intensity leads to a significant increase in the temperatures at the leading edge and the pressure-side wall. The temperature rises by approximately 20 K at the leading edge when TU = 20%. The influence of turbulence intensity on the pressure-side wall temperature is more significant than that on the suction side.

#### 3.2.3. Effect of manufactured vane profile deviation on the surface temperature.

The E3 engine NGV test specimen was 3D-printed. The impact holes, film-cooling holes, and pressure measurement holes were integrally modeled and printed simultaneously.

To determine the profile deviation of the manufactured vane, coordinate data for the guide vane surface should be obtained. Currently, methods such as optical measurement, coordinate measuring machine (CMM), or computed tomography (CT) scanning machines are typically employed to acquire the guide vane’s profile information. Optical measurement techniques, such as blue light scanners, can quickly obtain the point cloud of the entire vane or blade surface, although their accuracy is relatively low (~0.05 mm). In contrast, the accuracy of CMM is higher (~0.01 mm). However, capturing the entire surface shape requires multiple sections, making the process time-consuming. CT scanning can acquire data for both the outer surface and the inner cavity of the vane/blade. However, the measurement accuracy is influenced by the vane/blade material, and the process is both time-consuming and expensive. In this study, CMM was used to measure the shape of the 3D-printed vane, and the data was then used to reconstruct the entire vane surface. This reconstructed surface was compared with the designed one to quantify manufacturing deviations.

The reconstruction of vane surface mainly includes steps for smoothing measurement data, supplementing missing data, and 3D modeling from sectional data. Due to the presence of film-cooling holes and slots at the trailing edge, the CMM measurement data is neither smooth nor complete. Therefore, during the reconstruction process, curve-fitting tools are used to smooth out the sectional measurement data. After that, the section coordinate data need to be sorted from leading edge to trailing edge. When encountering missing data of the slot at the trailing edge, we can replenish the coordinate information with the designed section profile. Repeating the above step, we can achieve every measured section profile.

**Fig 19:**
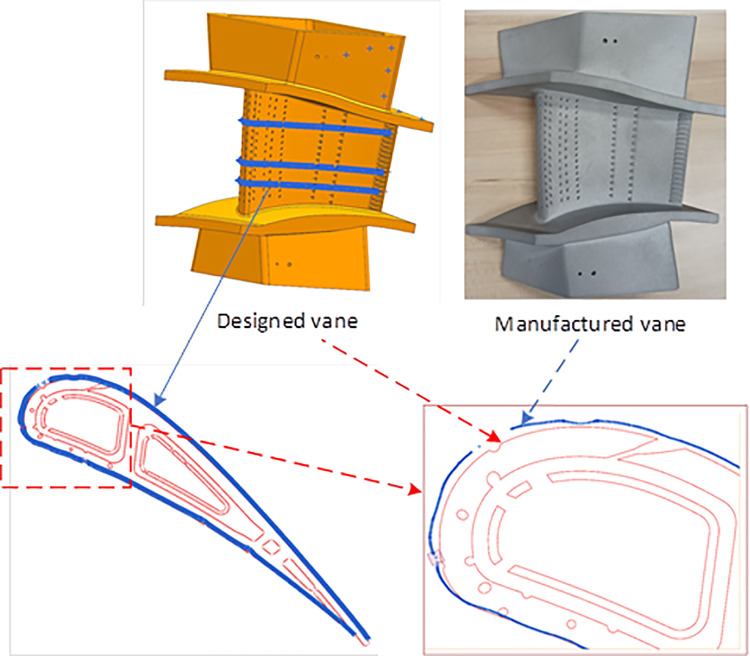
Comparison between the manufactured and designed vane.

In reality, the number of vane sections measured is generally limited. Therefore, an inverse procedure with the aerodynamic design program [31] which integrating the geometry design, aerodynamic simulation and blade stack into one software, is employed to generate the 3D profile of the vane or blade. By assuming no changes in the inner cavity, location of the film-cooling holes, and other structures, a model for the manufactured vane is generated. Fig 19 illustrates the comparison between the manufactured and designed vane profiles.

**Fig 20:**
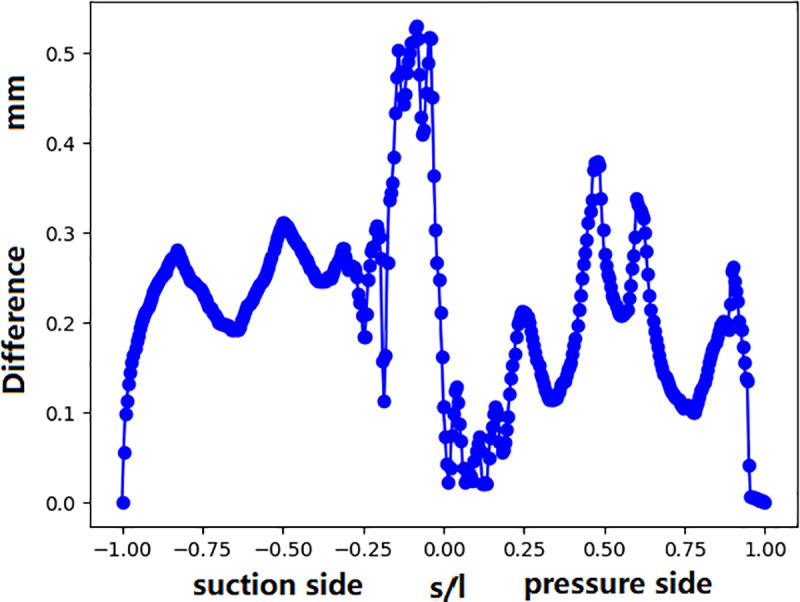
Profile difference between the manufactured and designed vanes (middle section).

Fig 20 presents the quantitative values of the deviation in the middle section profile between the manufactured and designed vanes. The maximum deviation at the leading-edge area can be up to 0.53 mm. In the mid-chordal region, the deviation ranges between 0.1 mm and 0.35 mm, with the lowest values observed at the trailing edge region.

Table 5 lists the computational mass flow rate results for one NGV passage in the previous computation. It compares the calculated mass flow rate for a single passage between the manufactured (UC6) and designed (UC0) vanes. The main-stream mass flow rate for UC6 is 2.9% smaller than that for UC0, primarily due to changes in the throat area. Correspondingly, the coolant flow rate for the front and aft cavities decreases by 2.3% and 2.5%, respectively, compared to the design vane.

**Table 5:** Mass flow rate for manufactured and designed vane (for single passage).

	Baseline (UC0)	Shape (UC6)	Difference %
Inlet flow rate, kg/s	0.4997	0.4854	-2.9%
Coolant mass flow rate in pre-cavity, kg/s	0.0218	0.0213	-2.3%
Coolant mass flow rate in aft-cavity, kg/s	0.0159	0.0155	-2.5%

**Fig 21:**
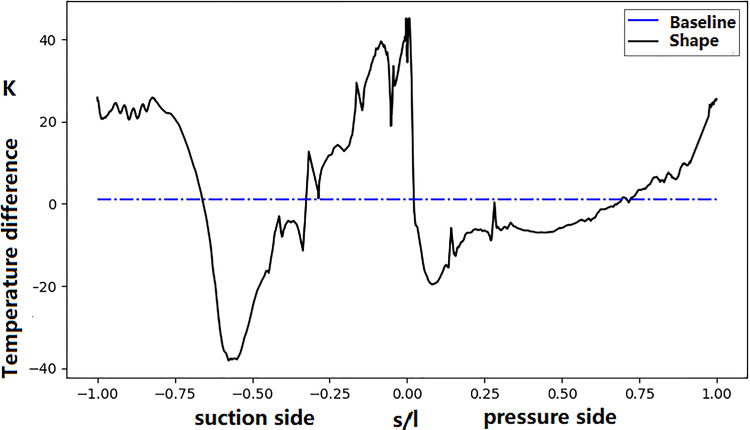
Effect of manufacturing deviation on NGV surface temperature.

The temperature difference between the designed and manufactured vanes is depicted in Fig 21. A notable temperature increase is observed at both the leading and trailing edges—the maximum temperature increase at the leading edge is approximately 40 K, and that at the trailing-edge area is approximately 20 K. To discover the reason, the streamline and turbulence kinetic energy distribution are plot.

**Fig 22:**
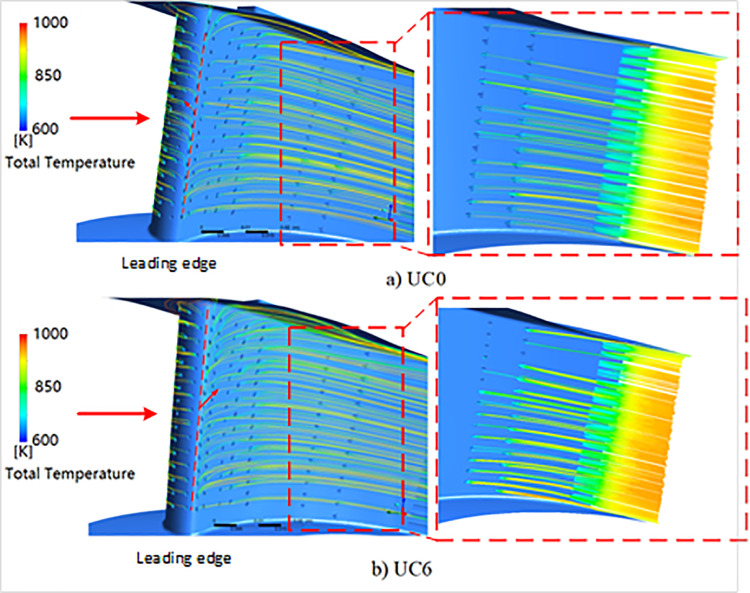
Comparison of the streamlines between the designed (a) and manufactured (b) NGV.

Fig 22 shows the streamlines of coolant outflow ejecting from the film-cooling holes. Significant profile deviation at the leading-edge results in a slightly change of the aerodynamic stagnation point. This causes the direction of the coolant outflow for the film-cooling holes near the stagnation point changing from the suction side to the pressure side. Consequently, there is a dramatic temperature improvement at the leading edge on suction side. Simultaneously, due to increased cooling air coverage, the surface temperature on the pressure side is noticeably lowered.

#### 3.2.4. Discussions.

**Fig 23:**
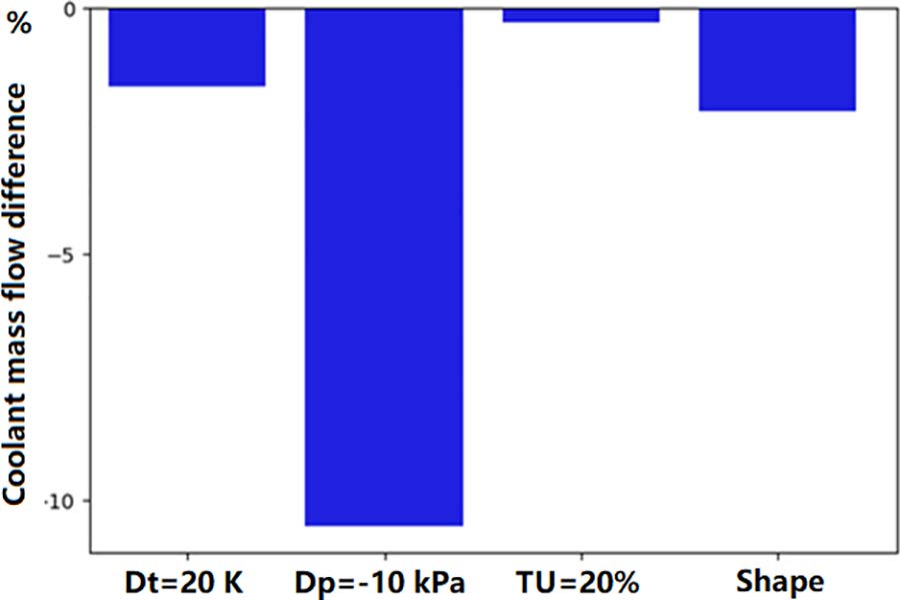
Effects on cooling air mass flow rate.

**Fig 24:**
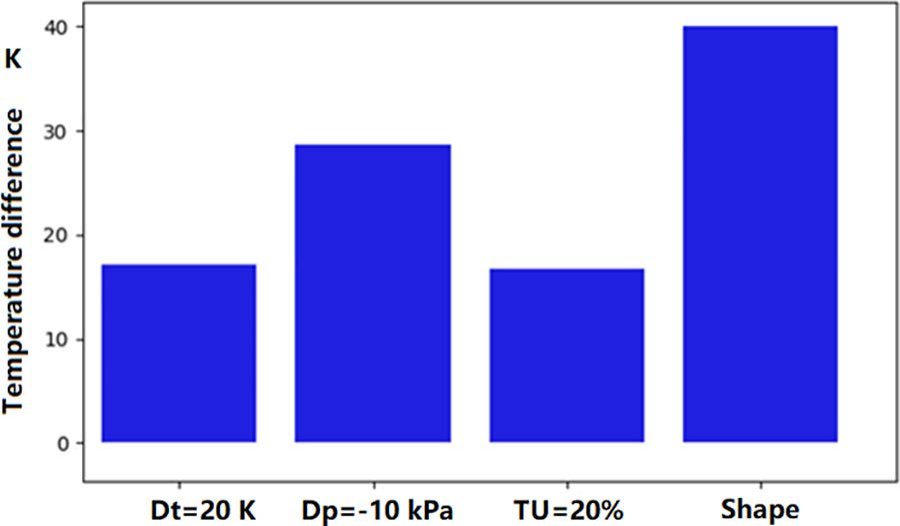
Effect on the NGV leading edge temperature.

**Fig 25:**
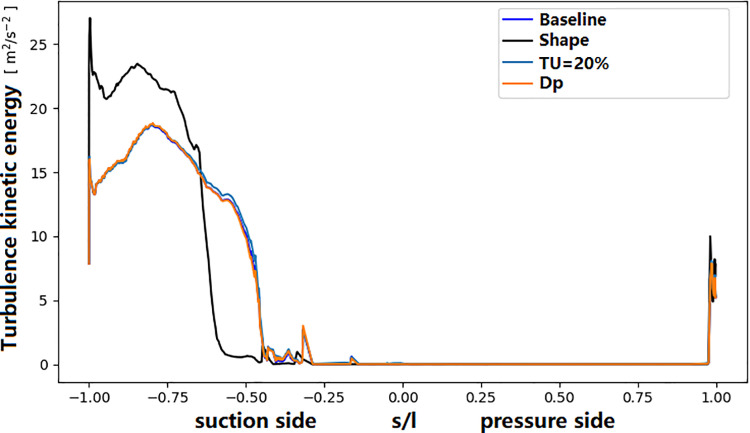
Effect on the turbulence kinetic energy along the NGV surface.

In the above analysis, we tried to find the reason of the significant deviation of the surface temperature distribution between simulation and experiment. From the above analyses, both the main-stream and coolant parameters, as well as manufacturing deviations, can impact the temperature of the turbine guide vane. In fact, these parameters have direct influence on the coolant mass flow rate. The sensitivity of mass flow rate to these parameters is shown in Fig 23. It can be seen that the deviation of coolant supply pressure has the most significant impact on the mass flow rate. Only 1% decreasing (10kPa) of the coolant pressure can cause the coolant mass flow rate decreasing by 10 percent. The shape deviation of the vane surface and the temperature increase (20K) have similar effect. The mainstream turbulence intensity has almost no influence on the mass flow rate. Regarding the surface temperature at the stagnation point (Fig 24), surface profile deviation(shape) has the greatest impact, causing a temperature increase of approximately 40 K. It not only causes the reduction of the coolant mass flow rate, but also the direction of film cooling flow. A reduction in the coolant supply pressure leads to a temperature increase of approximately 30 K, while the turbulence intensity (TU = 20%) and the coolant inlet temperature rise (Dt = 20 K) contribute to a temperature improvement of approximately 20 K. Fig 25 shows the distribution of the turbulence kinetic energy along the vane surface. It shows that the profile deviation (shape) causes the distribution of turbulence kinetic energy changing dramatically. The turbulence enhancement leads to the temperature increasement at the trailing edge of the middle section (as was shown in Fig 20).

However, in this study we have not considered the error caused by the turbulence model. In recent years, unsteady Reynolds-averaged Navier–Stokes, large eddy simulation, and direct numerical simulation models [32–34] have been developed dramatically. They can be employed to yield more accurate results. In addition, currently each influential factors changes individually. In the future, more uncertainty quantification methods can be used to investigate the combination effects of these factors.

## 4. Conclusions

In this paper, conjugate heat transfer analysis was conducted to study the aerodynamic and heat transfer performance of the E3 engine nozzle guide vane. Computational fluid dynamic analysis indicates that the shear stress transport turbulence model gives high accuracy in simulating the vane main-stream aerodynamic performance. However, when the simulated surface temperature of the vane was compared with the cooling test data, significant deviation of the surface temperature distribution was observed, including both the leading edge and the trailing edge of the suction side. Uncertainty analysis was conducted to identify the sources of these discrepancies, including the main-stream and coolant flow parameters as well as manufacturing deviations. Specifically, a significant deviation in the manufactured vane—up to 0.5 mm at the leading edge—alters the coolant flow direction in the leading-edge film-cooling holes, thereby affecting air film coverage and increasing the temperature near the stagnation point by approximately 40 K. In addition, it also changes the distribution of turbulence kinetic energy and causes the temperature at the trailing edge of the NGV increasing by approximately 20 K. Variations in coolant inlet pressure and temperature cause temperature increasing by 20 ~ 30 K. The increasing main-stream turbulence also influences wall heat flow and heat transfer, causing a surface temperature increase of approximately 20 K. Therefore, when conducting CHT analysis for turbine vane or blade, emphasis should be placed on the aerodynamic and manufacturing deviations that contribute to uncertainties of temperature.

However, 3D printing may not only cause the surface profile deviation, but the surface roughness inside the cooling channel. The surface roughness of the internal channel can not only enhance the heat transfer, but also the flow resistance which should be studied carefully in future studies.

### Nomenclature

**Table:** 

G_in_	=	Mass flow rate for turbine inlet
G_out_	=	Mass flow rate for turbine outlet
P* _in_	=	Total pressure of turbine inlet
T^*^_in_	=	Total temperature of turbine inlet
T^*^_out_	=	Total temperature of turbine outlet
K_w_	=	Mass flow ratio
$\pi_{T}$	=	Pressure ratio
$K_{T}$	=	Temperature ratio
$W_{C}$	=	Coolant mass flow rate of NGV
$W_{g}$	=	Mainstream mass flow rate of NGV
Pt_in	=	inlet total pressure of NGV
Ps_out	=	outlet static pressure of NGV
Tt_in	=	Mainstream inlet total temperature of NGV
$T_{C\_in}$	=	Coolant inlet total temperature of NGV
X/C	=	relative chord length
X	=	axial length from the surface point to the leading edge.
s	=	arc length from the surface point to the stagnation point, positive for pressure side and negative for suction side
l	=	arc length from the stagnation point to the trailing end point
s/l	=	relative arc length
W_25_	=	mass flow of high pressure compressor inlet
T_norm_	=	Normalized surface temperature

## Abbreviates

CHT: Conjugate Heat Transfer
E3: Energy Efficiency Engine
TIT: Turbine Inlet Temperature
NGV: Nozzle Guide Vane
HPT: High Pressure Turbine
CMM: Coordinate Measuring Machine
